# Rapid and Localized Mechanical Stimulation and Adhesion Assay: TRPM7 Involvement in Calcium Signaling and Cell Adhesion

**DOI:** 10.1371/journal.pone.0126440

**Published:** 2015-05-06

**Authors:** Wagner Shin Nishitani, Adriano Mesquita Alencar, Yingxiao Wang

**Affiliations:** 1 Department of Bioengineering & Beckman Institute for Advanced Science and Technology, Center for Biophysics and Computational Biology, Institute for Genomic Biology, University of Illinois, Urbana-Champaign, Urbana, Illinois, 61801, United States of America; 2 Department of Mechanical Science & Engineering, Integrative and Molecular Physiology, Center for Biophysics and Computational Biology, Institute for Genomic Biology, University of Illinois, Urbana-Champaign, Urbana, Illinois, 61801, United States of America; 3 Instituto de Física, Universidade de São Paulo, São Paulo, São Paulo, Brazil; 4 Department of Bioengineering & Institute of Engineering in Medicine, University of California, San Diego, La Jolla, California, 92093, United States of America; Irvine, UNITED STATES

## Abstract

A cell mechanical stimulation equipment, based on cell substrate deformation, and a more sensitive method for measuring adhesion of cells were developed. A probe, precisely positioned close to the cell, was capable of a vertical localized mechanical stimulation with a temporal frequency of 207 Hz, and strain magnitude of 50%. This setup was characterized and used to probe the response of Human Umbilical Endothelial Vein Cells (HUVECs) in terms of calcium signaling. The intracellular calcium ion concentration was measured by the genetically encoded Cameleon biosensor, with the Transient Receptor Potential cation channel, subfamily M, member 7 (TRPM7) expression inhibited. As TRPM7 expression also regulates adhesion, a relatively simple method for measuring adhesion of cells was also developed, tested and used to study the effect of adhesion alone. Three adhesion conditions of HUVECs on polyacrylamide gel dishes were compared. In the first condition, the substrate is fully treated with Sulfo-SANPAH crosslinking and fibronectin. The other two conditions had increasingly reduced adhesion: partially treated (only coated with fibronectin, with no use of Sulfo-SANPAH, at 5% of the normal amount) and non-treated polyacrylamide gels. The cells showed adhesion and calcium response to the mechanical stimulation correlated to the degree of gel treatment: highest for fully treated gels and lowest for non-treated ones. TRPM7 inhibition by siRNA on HUVECs caused an increase in adhesion relative to control (no siRNA treatment) and non-targeting siRNA, but a decrease to 80% of calcium response relative to non-targeting siRNA which confirms the important role of TRPM7 in mechanotransduction despite the increase in adhesion.

## Introduction

A deeper understanding of physiology is possible by understanding the living cell responses to changes in the mechanical environment, composed by mechanical properties of the substrate and environmental mechanical forces. Indeed, the mechanical properties of the substrate determine the fate of stem cells affecting embryogenesis [1] and cell migration [2]. Environmental mechanical forces, such as fluid shear stress, have been related to the development of atherosclerosis [3,4], thrombosis and hypertension [4]. Excessive cyclic mechanical stretching of microvascular endothelial cells in the lung tissue of patients may cause further damage, denominated ventilator-induced lung injury (VILI) [5,6]. However, the mechanism by which cells perceive these mechanical forces and coordinate intracellular molecular signals are yet to be fully understood. To do so, one should be able to deliver mechanical stimulation in a controlled way and observe the cell response.

Many techniques are available to mechanically stimulate cells, including the patch-clamp used to study ion channels. In this technique, the suction forces in the patch-clamp can be adjusted to induce conformational changes of ion channels and the alteration of their properties [7]. Cells can also be mechanically stimulated through fluid shear stress in a flow chamber [3,8] or by stretching a flexible substrate where the cells are plated on, such as a silicone membrane [9]. Another way to deliver mechanical forces is through laser tweezers [10], where a laser trap system is able to apply and measure forces on a bead in the order of piconewtons. Beads previously attached to the membrane can also be used to apply force from a glass probe [11] or simply from a magnetic field, if the beads are magnetized [12,13]. Glass probes were also shown to touch the cells and evoke calcium signaling [14]. Although there are several techniques to mechanically stimulate cells, most of them are either strain or temporal frequency limited. Stronger cell responses have been correlated to high temporal frequency mechanical stimulation [3,15], such as observed during cell injury following mechanically traumatic events on tissue. These traumatic event studies also require higher strains, in the order of 30% [6,15]. Being able to simultaneously deliver strain and high temporal frequency mechanical stimulation enlarges the envelope of possibilities.

Once the mechanical stimulation is delivered, it is necessary for the cell to translate it into biochemical signaling. Mechanosensitive (or stretch-activated) ion channels on the plasma membrane are part of the mechanotransduction cellular apparatus, translating mechanical stretches into an ion current through the membrane [16]. Some of these mechanosensitive ion channels are the complexes formed by MEC-4, MEC-10, MEC-2 and MEC-6, several members from TRP subfamilies [17], and Piezo1 and Piezo2 [17,18]. These are especially important when investigating mechanotransduction in live cells. One member of the TRP subfamilies, the Transient Receptor Potential cation channel, subfamily M, member 7, also known as TRPM7, is ubiquitously expressed [19], possesses a kinase domain [20,21], and potentially has a role in hypertension control in vascular systems [19]. In addition to its involvement in mechanotransduction [19,21], TRPM7 expression inhibition or overexpression modulates adhesion in some cells types [20,22]. Thus, in addition to the study of the mechanotransduction itself, it is important to understand the impact of TRPM7 expression on adhesion of HUVECs in order to draw conclusions.

Regarding the biochemical cell response to mechanical stimuli, one important molecule to monitor is the calcium ion Ca^2+^. The intracellular calcium ion concentration [Ca^2+^]_i_ has been shown to play a crucial role in a variety of physiological signaling pathways and to be sensitive to mechanical cues. In fact, shear stress can cause the rise of [Ca^2+^]_i_, which is involved in many signaling pathways, like the production of nitric oxide [8]. When actin stress fibers are directly stretched by optical tweezers or when fibronectin-coated beads previously attached to the apical surface of the cell are moved, stress-activated calcium channels are opened [11]. Five minutes of local deformation of NIH3T3 cell substrate can change cell orientation and increase both [Ca^2+^]_i_ and cell traction forces [23]. It has also been shown that an increase in [Ca^2+^]_i_ is propagated among astroglial cells after mechanical stimulation by a glass probe touch [14]. On the other way, it is well known that calcium ion is involved in actomyosin contractility, regulating the stress fiber tension, and can change other mechanical properties of cells, such as the stiffness of apical surfaces in HUVECs [12].

To visualize [Ca^2+^]_i_ in real time with high spatiotemporal resolution, one could use calcium dyes, such as Fura-2 [24]. Another way is to use genetically encoded molecular biosensors. For example, Cameleon, a Fluorescence Resonance Energy Transfer (FRET) biosensor based on the interacting pair Calmodulin and M13. These biosensors allow the detection of [Ca^2+^]_i_ with high precision [25,26], and have the advantage over calcium dyes of targeting subcellular regions or compartments according to their design [27]. We have previously discovered a FRET pair, ECFP and YPet, which allows a high sensitivity for the detection of a variety of molecular activities [13,27]. The FRET pair in Cameleon was also replaced by ECFP and YPet and applied throughout this work to monitor the mechanical-force-induced calcium signaling.

Thus, in this work, an equipment setup was characterized and used to probe the HUVEC response to mechanical stimulation. The equipment was capable of localized mechanical stimulation and relatively high temporal frequency and strain magnitude. It was based on a vertical vibration of the probe, different from the lateral vibration used in our previous article [28]. The vertical vibration optimizes the use of the mechanical energy, focusing it into a smaller area. This equipment also has a simpler design, easier to be reproduced. HUVECs were seeded on substrate with different adhesions or had their TRPM7 channels inhibited before being mechanically stimulated. A more sensitive method for measuring adhesion of cells was also developed to assess the adhesion conditions of HUVEC on the different substrates and the effect of TRPM7 expression modulations. Adhesion was then compared to calcium signaling in response to the mechanical stimulation. The cells had their calcium response to mechanical stimulation measured by the Cameleon biosensor.

## Methods

### Cell culture

Human umbilical vein endothelial cells (HUVECs) were purchased from Cell Applications (San Diego, CA). The cells were cultured in 100x20 or 60x15 mm cell culture dishes (Corning, 430167 and 430166) in 5% CO_2_ at 37°C and passaged when achieved 80% confluency or more. Medium was changed every other day or when the confluency reached 60% or more. The cells were used up to 16 doublings, the minimum number of doublings guaranteed by Cell Applications. The growth medium used was Endothelial Cell Growth Medium (Cell Applications, 211–500).

### Genetically encoded FRET biosensor

A genetically encoded FRET biosensor based on ECFP and YPet was used to monitor [Ca^2+^]_i_ as previously described [25,26]. All cells used for assessment of response to mechanical stimulation were transfected with this biosensor. The method of choice for delivering the DNA into the cell was the adenovirus infection (Adeno-X Expression System 1, Clontech), in which the biosensor plasmid was incorporated. For the most basic infection, with no prior electroporation preformed, the cells were passaged from a 80 to 90% confluent dish into a 35x10 mm glass-bottom dish between 16 and 18 hours before the infection. The cells were then infected with the adenovirus carrying the FRET biosensor. If electroporated cells were to be infected, they were done so 2 hours after electroporation and after a change of growth medium to remove detached cells. In both cases (simple infection or infection after electroporation), the infected medium was changed to normal growth medium after 4 hours of incubation. In the next day, the infected cells were passaged to polyacrylamide gel dishes at a convenient count for the experiment: 1,000 to 3,000 cells/dish for mechanical stimulation or 3,000 to 6,000 cells/dish for adhesion test. These gel dishes were ready for the vibration experiments about 20 hours later.

### Polyacrylamide gel preparation

The polyacrylamide gel dishes were prepared according to a well-established protocol [2] with the following reagents: Acrylamide at 8% (40% solution stock, Bio-Rad, 161–0140), Bis at 0.13% (2% solution stock, Bio-Rad, 161–0142), TEMED at 1:2,000 (Bio-Rad, 161–0801) and 10% w/v Amonium Persulfate at 1:200 (Bio-Rad, 161–0700) in 10 mM HEPES buffer (Sigma, H4034). The stiffness of the gels was 20 kPa approximately [29]. The gels were cast on 35x10 mm glass-bottom dishes with a 14 mm well using a 12 mm round cover glass (Fisher, 12-545-80) to shape the droplet of gel solution. The volume of the gel solution droplet was calculated to allow an average thickness of 90 μm.

Before HUVEC were plated on polyacrylamide gels, the gels were activated with Sulfo-SANPAH (Thermo Scientific, 22589) and coated with fibronectin (Sigma, F1141), unless noted otherwise. For the activation with Sulfo-SANPAH, each gel was covered with 0.1 mg of Sulfo-SANPAH diluted in 200 μl of 100 mM HEPES buffer, positioned around 5 cm away from the UV light source in the biosafety cabinet, exposed to UV light for 6 minutes, rinsed with 100mM HEPES buffer and placed on an orbital shaker for 10 minutes. Then, the gel dishes were rinsed with Phosphate Buffered Saline (PBS) (Sigma, D5652) and soaked in it for 5 minutes on an orbital shaker. After the PBS was removed, each gel was incubated in a solution with 3 μg of fibronectin diluted in PBS for 4 hours. The fibronectin concentration on the dish was about 2 μg/cm^2^. After the incubation, the gel dishes were sterilized by UV exposure in the biosafety cabinet for 20 minutes and rinsed with PBS. The gels were soaked in growth medium 15 minutes before the cells are plated on. The cells should be plated on the gels around 24 hours before the experiments.

To obtain the gel with beads, a second layer of polyacrylamide with embedded beads was prepared on top of the clear gel layer in a similar fashion [30], resulting in almost all of the beads in a single layer and at the same focal plane when observed through a 40x/0.75 objective. In brief, the solution for the polyacrylamide was mixed with a 1 μm-bead suspension (Invitrogen, F-8821) at 1:250 and a small amount (1~2 μl) was applied on top of the clear gel. Upon the placement of the cover glass, a thin film of solution with beads would form the bead-embedded second gel layer on top of the pre-cast gel. As long as the pre-cast clear gel is smaller than the cover glass, capillarity will force the excess of solution with beads to the side, allowing the formation of the thin layer.

### Fibronectin quantification

To verify the amount of fibronectin coated on the gel substrates, fibronectin from bovine plasma in powder (Sigma, F4759) was conjugated with NHS-Rhodamine (Thermo Scientific, 46406). First, the fibronectin was dissolved in borate buffer (100 mM boric acid and 7.25 mM sodium tetraborate in water, pH 8.5) and NHS-Rhodamine, in Dimethylformamide (DMF) at 10 mg/ml. NHS-Rhodamine was mixed to the dissolved fibronectin at a molar ratio of 10 (NHS-Rhodamine to fibronectin) and incubated at room temperature for 1 hour. Then, the nonconjugated rhodamine was removed by dialysis overnight at 4°C. This fibronectin conjugated to rhodamine was used to coat gel substrate and each gel substrate was imaged around 25 times at random locations. The average fluorescence intensity was calculated for each image taken.

### Electroporation

Due to the difficulty of transfecting DNA or RNA into HUVECs, electroporation was used for introducing siRNA into these cells. The siRNAs transfected were Human TRPM7 (Dharmacon, L-005393-00) and Non-targeting pool (Dharmacon, D-001810-10-05). As in the case of infection by adenovirus, the cells were passaged from a 80 to 90% confluent dish into 100x20 or 60x15 mm cell culture dishes between 16 and 18 hours before the electroporation. At the electroporation time, approximately 120,000 cells were resuspended in Gene Pulser electroporation buffer (Bio-rad, 165–2676) and placed in a Gene Pulser cuvette, 0.2 cm electrode gap (Bio-rad, 165–2082), along with siRNA at 100 nM or 200 nM. The electroporation of HUVECs was then conducted in a Gene Pulser Xcell Total System (Bio-Rad, 165–2660), with a single pulse of a square wave at 150 V for 20 ms. The electroporated cells were plated on a 35x10 mm cell culture dish (Corning, 430165). Experiments were conducted 2 days after electroporation.

### Western blot

Cells electroporated with siRNA were lysated after two days of incubation and had their TRPM7 (210 kDa) and Glyceraldehyde 3-Phosphate Dehydrogenase (GAPDH, 37 kDa) expression examined by Western blot to verify the inhibition of TRPM7 expression by siRNA. 16 μg of total proteins in 2% SDS loading buffer were separated by polyacrylamide gel (10% mixture) electrophoresis, along with a pre-stained protein standard (Invitrogen, 10748010), and transferred to a nitrocellulose membrane (Bio-Rad, 162–0115). After blocking unspecific binding with 5% milk for one hour, the membrane was cut and incubated overnight at 4°C on a rocker with primary antibodies: 1:1000 goat anti-TRPM7 (abcam, ab729) for the top part (>60 kDa) and 1:1000 goat anti-GAPDH (Santa Cruz) for the bottom part (<60 kDa). Then, the membrane was incubated in the horseradish peroxidase-conjugated secondary antibody donkey anti-goat immunoglobulin (Santa Cruz, SC-2020) at 1:5000 for one hour at room temperature on a shaker. The protein expression was observed with SuperSignal West Femto enhanced chemiluminescence (ECL) substrate (Thermo Scientific, 34094). Each part of the membrane was photographed with the necessary exposure to allow a clear image but avoid saturation, according to the amount of expression. ImageJ was used for background subtraction and the integration of chemiluminescence from TRPM7, which was normalized by the GAPDH integration.

### Adhesion assay

**Fig 1:**
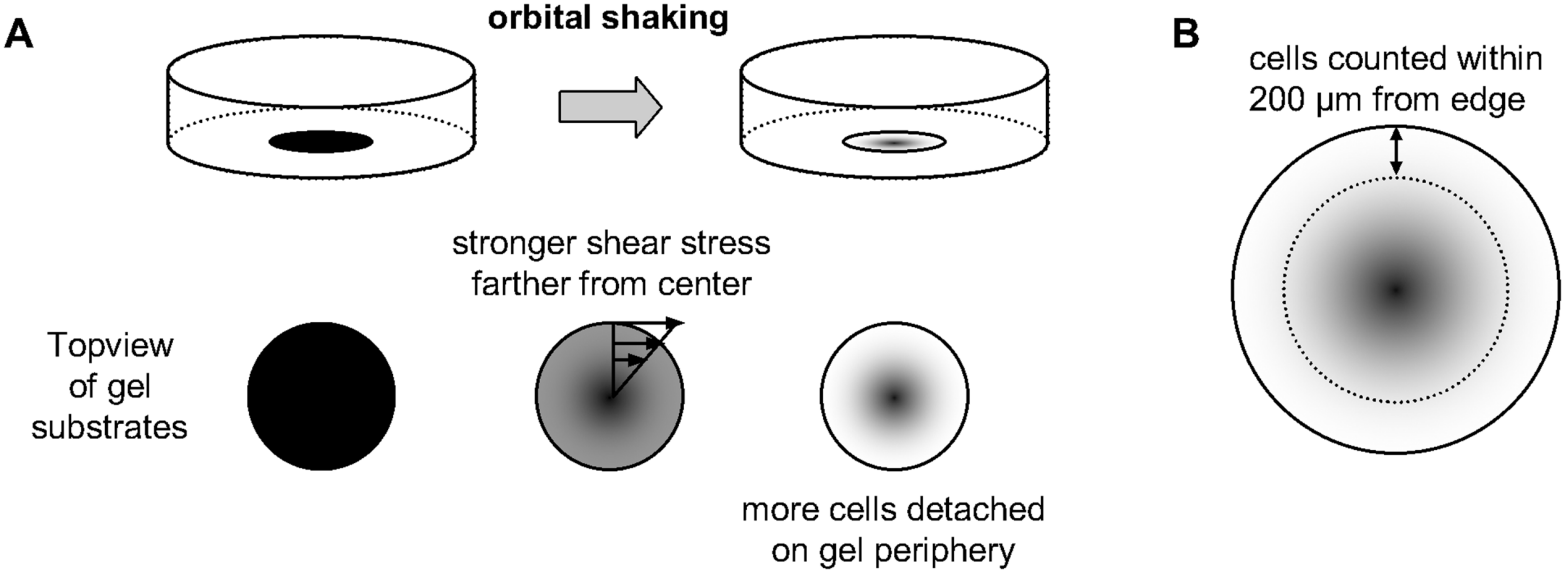
Uneven shear stress in adhesion assay and cell counting method. (A) The nature of the orbital shaker causes a stronger shear stress at the periphery of the gel, causing a stronger cell detachment at these regions (denoted by the lighter color at the periphery in the diagram). (B) Cells are counted only at the periphery, from the edge up to 200 μm towards the center, before and after the shaking.

The main characteristic of this assay was to use an orbital shaker to create a shear stress to detach the cells on gel and count the remaining cells only at the periphery of the gel, before and after the cell culture dish was shaken. Due to the working principle of the orbital shaker, the fluid flow in the dish is uneven, imposing a stronger shear stress farther from the center of the dish(Fig 1A). Thus, all the cell countings, before and after the shaking, were done only at the periphery of the gel, to increase the sensitivity of the method(Fig 1B).

First of all, before any shaking was done, HUVECs were counted from the periphery of the polyacrylamide gel, from the edge to 200 μm towards its center. Around 20 screenshots were taken randomly and the average of cells per screenshot was calculated. Growth medium, previously equilibrated in 5% CO_2_ incubator for at least 30 minutes, were added to the gel dishes to a final volume of 4 ml in each one. The dishes were then placed on an orbital shaker (Barnstead 2314) and shaken 3 times at top speed (about 220 rpm) for 10 seconds, with 5 seconds of interval between shakings. The growth medium was changed to remove detached cells and the attached ones were counted in another set of around 20 screenshots at the periphery per dish. For each screenshot taken after the shakings, an attachment ratio of the cells (*AttRatio*) was calculated:

$$DetRatio=\frac{C_{after}}{{\bar{C}}_{before}}\;,$$

where *C*_*after*_ is the cell count of the screenshot after shaking and ${\bar{C}}_{before}$ is the average cell count per screenshot for the same dish before shaking.

All attachment ratios for one condition were compared to the attachment ratios for another condition to assess the relative adhesion between them.

### Mechanical stimulation of HUVEC

**Fig 2:**
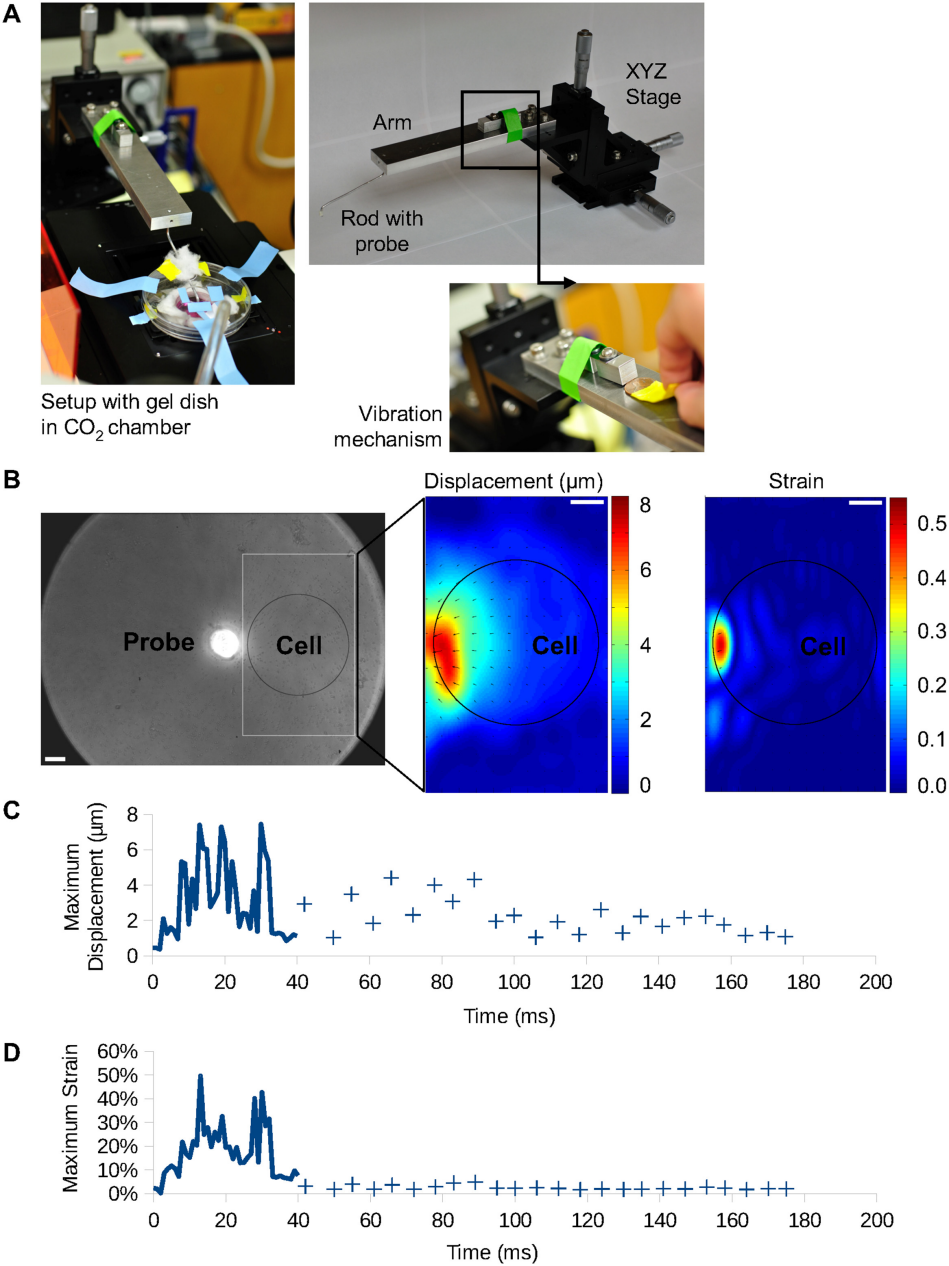
Mechanical stimulation equipment and vibration characterization. (A) Pictures of the vibration stimulation equipment with different components: XYZ stage, with micrometers attached, aluminum arm and aluminum rod with probe (top right panel); vibration mechanism and spacer ready to trigger the vibration (bottom right panel); setup with the aluminum rod already inserted in the aperture of the CO_2_ chamber and with probe positioned on the gel (left panel). (B) The left image shows the probe and gel substrate with 1 μm beads embedded. The right images show a typical displacement (in μm) and strain maps, with the cold and hot color representing the small and large displacement/strain, respectively. In both colormaps, the vectors are the displacements on the substrate. The black circle represents the typical position of a cell (~125 μm of diameter) during stimulation. The bars represent 25 μm. (C) Maximum displacement of the gel over time. (D) Maximum strain of the gel over time. For both graphs, the data points are plotted with 1 ms interval for the first 40 ms of vibration and, until 180 ms, for the frame with largest probe displacement in each vibration period.

The stimulation equipment was designed to position the probe tip on the surface of the substrate gel and generate a vibration on the gel where the cells were seeded. The probe tip consists of a glass capillary prepared by a micropipette puller with an approximate diameter of 35 μm in average. The probe tip was attached to an aluminum arm. The arm was then attached to an XYZ linear stage (Thor Labs, PT3). The XYZ stage was used to position the probe tip 6 μm away from the cell edge, barely touching the gel substrate. Once positioned, the vibration mechanism was activated, locally vibrating the substrate and stimulating the cell. The vibration mechanism was composed of two small aluminum blocks attached to each other by a spring steel sheet. One block was attached to the top surface of the aluminum arm while the other was free but in contact with the same plane of the arm. The vibration mechanism was triggered by removing a spacer between the free aluminum block and the arm, allowing the collision between them. This collision initiated a vibration which was mainly vertical due to the position of the vibration mechanism. The vibration propagated through the structure and was transmitted to the gel substrate by the small aluminum rod and probe tip(Fig 2A). Due to the angle of the rod relative to the gel substrate plane, the probe tip will also vibrate at an angle, not completely orthogonal to this plane. Thus, considering the position of the probe tip and the cell, the gel was slightly pulled away from the cell whenever the probe tip oscillated downward. The vibration frequency of the probe tip was estimated by analyzing the natural frequency of the two main structural components of the system: the arm and the small rod. The simplified model of a cantilever beam anchored on one end allowed an estimation of the natural frequency of both parts. By modeling the arm as an aluminum bar 16.7x1.25x3.8 cm, the natural frequency of the first mode of vibration was approximately 1,100 Hz. The small rod, modeled as a cylinder 8 cm long with a radius of 1 mm, had a natural frequency of the first mode of vibration of 220 Hz. The vibration frequency of the probe tip would be a composition of both vibrations and, due to the difference in stiffness, it was expected for the small rod to be the main contributor. Taking into account the mass added to the rod tip with the attachment of the glass probe, the vibration frequency would be lowered. Thus, the vibration frequency of the component with higher displacement would be lower than 220 Hz but with the same order of magnitude.

For all samples, the cells were imaged for a few minutes to record the basal FRET ratio. The imaging was then paused to place the probe tip in position and resumed immediately after the mechanical stimulation was triggered and the probe tip lifted from the gel surface.

To keep the cell environment stable during imaging and vibration stimulation, a chamber was designed to allow the access of the probe to the cell dish as well as the constant entry of pre-mixed and humidified 5% CO_2_ (along with 10% O_2_ and 85% N_2_) (Fig 2A). A controlled heater (Nevtek ASI 400) was connected to maintain the temperature around 37°C throughout the experiment in the chamber.

### Imaging

A Zeiss fluorescence microscope equipped with an oil-immersed 40x/1.3 objective was controlled by a computer through the software MetaFluor 6.3r7 (Molecular Devices) to obtain the live cell images of the biosensor FRET signals. A xenon arc lamp excited the donor fluorophore ECFP by using a 420/20 nm filter. A 475/40 nm and 535/25 nm filters were used to observe the emissions from the donor (ECFP) and acceptor (YPet), respectively. A complete set of images (brightfield, donor and acceptor fluorescence images) was acquired every 30 seconds before stimulation, increasing this frequency up to 5 seconds per frame after the mechanical stimulation, in order to capture the precise temporal character of the FRET response.

For the adhesion assay, the same Zeiss microscope was used for phase contrast with a long working distance 10x/0.25 objective.

For the fibronectin quantification, the Zeiss microscope observed rhodamine fluorescence with a long working distance 10x/0.25 objective. A xenon arc lamp excited rhodamine by using a 550/10 nm filter. A 575/15 nm filter was used to observe the emission from rhodamine.

For the vibration characterization, a fast camera (Vision Research Phantom v9.1) with 1,000 frames/second capacity was coupled to a phase contrast microscope with a 40x/0.75 objective to image the trajectory of the probe tip and the displacement generated on the flexible substrate gel containing beads. An extra halogen light was employed to obtain enough illumination for high frame rates. These experiments were conducted at the Imaging Technology Group at the Beckman Institute for Advanced Science and Technology, University of Illinois at Urbana-Champaign.

### Postexperimental imaging analysis

**Fig 3:**
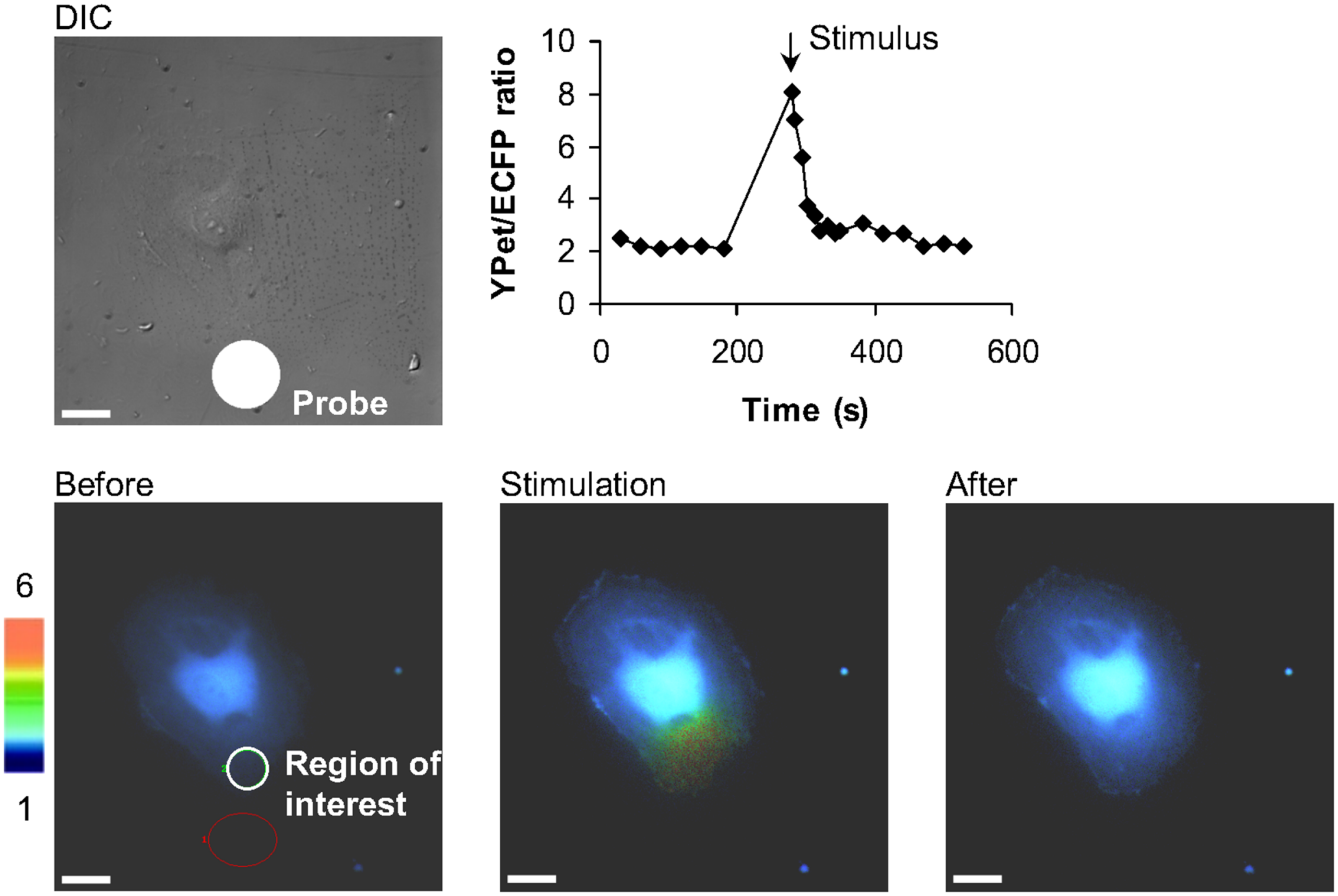
Typical calcium response upon mechanical vibration stimulation. Typically, the cell responds with a rise in [Ca^2+^]_i_ occurring mostly at a region close to the stimulation site. The DIC images show the location of the probe and the cell under stimulus. The time course represents the [Ca^2+^]_i_ at the region of interest. The color images represent the fluorescence emission ratio of YPet/ECFP from the biosensor before stimulation, immediately after stimulation, and after [Ca^2+^]_i_ re-stabilized. The bars represent 25 μm.

Live cell images of the biosensors were captured and analyzed by MetaFluor 6.3r7. The fluorescence of the calcium biosensor FRET pair was observed in a circular region of interest, with 50 pixels of diameter, as close as possible to the area stimulated by the probe (Fig 3). The FRET ratio, directly related to [Ca^2+^]_i_, was calculated by dividing the average intensity of YPet by that of ECFP. A region not covered by the cells and close to the probe tip was selected to assess the background signal, which was subtracted from the image. The change in [Ca^2+^]_i_ reported by the biosensor after mechanical stimulation was quantified as a relative difference (*RelDiff*) to basal measurements (before stimulation):

$$RelDiff=\frac{maxRatio-basalRatio}{basalRatio}\;,$$

where *maxRatio* is the maximum Ypet/ECFP ratio after stimulation and *basalRatio* is the average Ypet/ECFP ratio before stimulation.

To characterize the vibration, images from the fast camera were analyzed using an ImageJ (available at http://rsb.info.nih.gov/ij; developed by Wayne Rasband, National Institutes of Health, Bethesda, MD) plugin, bUnwarpJ [31]. This plugin was used to compare the position of the beads between two frames: a frame displaced by the probe motion and a reference frame with the probe merely positioned but not moving. Matlab (MathWorks) was applied to reconstruct the displacement map obtained from bUnwarpJ, plotting it as a colormap combined with the vectors for a convenient and clear understanding of the data. A colormap for the strain at each point was also plotted, with vectors denoting the displacement. The total strain E was calculated as:

$$E=\sqrt{E_{x}{}^{2}+E_{y}{}^{2}}\;,$$

where *E*_*x*_ and *E*_*y*_ are respectively the strain in *x* and *y* axis, or

$$E_{x}=\frac{du_{x}}{dx}\;\text{and}\;E_{y}=\frac{du_{y}}{dy}\;,$$

where *u*_*x*_ and *u*_*y*_ are the displacements in *x* and *y* axis, with each derivative numerically calculated by finite differences (2-point estimation for the boundaries, 3-point estimation for interior points).

### Statistical analysis

The p-values were calculated by two-tailed Student's t-test for the analysis of two sets of samples for both response to mechanical stimulation or adhesion assays. Statistical significance was defined as p<0.05.

## Results

### Characterization of mechanical stimulation

To characterize the deformation caused on the polyacrylamide gel substrate by the stimulation equipment, gel with beads were imaged with a fast camera at different thickness: 50 μm, 75 μm, 100 μm and 125 μm. The vibration frequencies observed in the substrate deformation were in average 207±2 Hz. In average, the maximum displacement observed was 8.8±2.4 μm and the maximum strain, 50±15%. Both displacement and strain were stronger when closer to the stimulation site, as expected. The vibration frequency and maximum deformation and strain observed for each substrate thickness are shown in Table 1. The most representative thickness is 125 μm, as most of the samples were stimulated on a substrate around or thicker than 100 μm. The gel with positioned probe tip and the displacement and strain maps of the frame with their respective maximum, for the 125 μm-thick gel are shown in Fig 2B and S1 Video, S2 Video and S3 Video. Graphs of maximum displacement and strain of the gel over time are shown in Fig 2C and 2D. In both graphs, the data points are plotted with 1 ms interval for the first 40 ms of vibration and, until 180 ms, for the frame with largest probe displacement in each vibration period. While it is not possible to directly observe the probe vertical motion, the frame with the largest probe displacement in a period of vibration was determined as the frame with the largest displacement for the beads closer to the probe.

**Table 1:** Displacement, strain and frequency of vibration for each gel thickness.

Thickness (μm)	Max Displacement (μm)	Max Strain (%)	Frequency (Hz)
50	10.0	55	211
75	11.6	65	206
100	6.2	30	206
125	7.5	50	206

For the experiments using mechanical stimulation, the control samples were HUVEC infected with Cameleon biosensor and seeded on polyacrylamide gel substrates. These gel substrates were treated with Sulfo-SANPAH and coated with the normal amount of fibronectin. The calcium response to mechanical stimulation consists in most cases of a transient increase in [Ca^2+^]_i_ in the vicinity of the probe tip, with weaker response at farther distances from the stimulated region (Fig 3, S4 Video).

### Lower substrate adhesion inhibits response to stimulation

**Fig 4:**
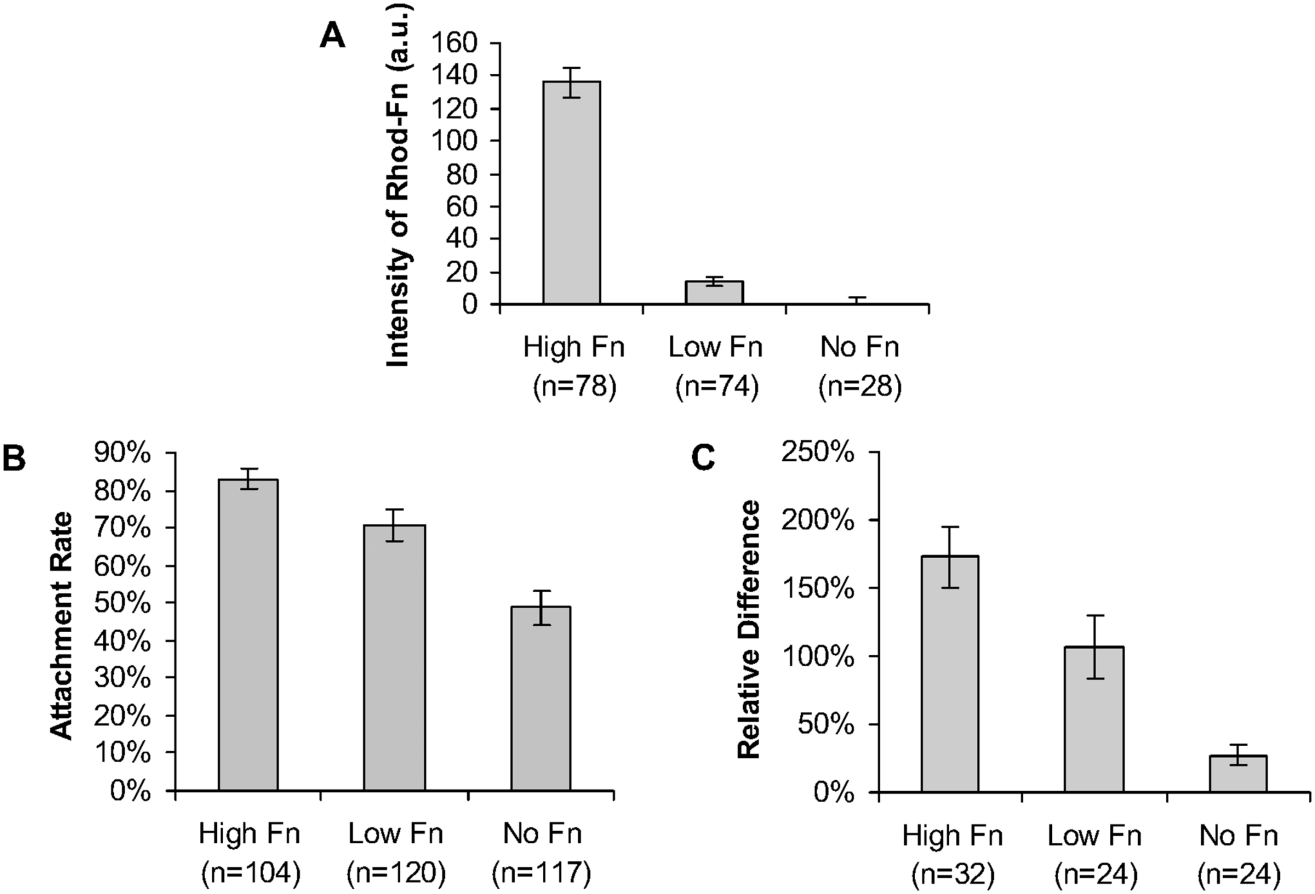
Lower substrate adhesion inhibits response to stimulation. Three different gel substrate treatments were used to assess the effect of adhesion on the calcium response to mechanical stimulation: regularly treated polyacrylamide gel substrates ("High Fn"), no Sulfo-SANPAH activation but coating with fibronectin at 5% of the normal amount per dish ("Low Fn"), no Sulfo-SANPAH and no fibronectin coating ("No Fn"). (A) Average intensities of rhodamine-labeled fibronectin of different gel substrates. All of them are statistically different among themselves (High Fn and Low Fn: p = 3x10^-21^, High Fn and No Fn: p = 2x10^-23^, Low Fn and No Fn: p = 0.007; Student's t-test). (B) Attachment ratios of HUVECs seeded on different gel substrates. All of them are statistically different among themselves (High Fn and Low Fn: p = 0.02, High Fn and No Fn: p = 7x10^-10^, Low Fn and No Fn: p = 0.0005; Student's t-test) (C) Relative difference of the biosensor fluorescence ratio of mechanically stimulated HUVECs seeded on different gel substrates. All of them are statistically different among themselves (High Fn and Low Fn: p = 0.04, High Fn and No Fn: p = 4x10^-7^, Low Fn and No Fn: p = 0.003; Student's t-test). The error bar on the bar graphs are the respective standard error of the samples.

Along with the regularly treated polyacrylamide gel substrates (“High Fn” dishes), other two types were also prepared to assess the effect of substrate adhesion on the response to mechanical stimulation, none of them with activation by Sulfo-SANPAH: one coated with fibronectin at 0.15 μg per dish, or 0.11 μg/cm^2^, (“Low Fn” dishes) and another with no coating of fibronectin (“No Fn” dishes). To assess the relative amount of fibronectin for each gel substrate treatment, rhodamine-labeled fibronectin was used in the treatment of 3 “High Fn” dishes and 3 “Low Fn” dishes. One “No Fn” dish was used as control and the average autofluorescence in this dish was subtracted from the average fluorescence of the other two treatments. Imaging of rhodamine-labeled fibronectin showed a difference of one order of magnitude between the average fluorescence in “High Fn” and “Low Fn” dishes. There was statistically significant difference among the fluorescence recorded for each of the three substrate conditions in this fibronectin quantification (High Fn and Low Fn: p = 3x10^-21^, High Fn and No Fn: p = 2x10^-23^, Low Fn and No Fn: p = 0.007; Student's t-test) (Fig 4A). To confirm the difference in adhesion among these conditions, the adhesion assay showed more cells still attached to the gel substrate when fully treated (“High Fn” dishes, with Sulfo-SANPAH activation and fibronectin coating) than when no Sulfo-SANPAH activation was done. “Low Fn” dishes showed also more attachment than “No Fn” dishes. There was statistically significant difference among all three substrate conditions in the adhesion assay (High Fn and Low Fn: p = 0.02, High Fn and No Fn: p = 7x10^-10^, Low Fn and No Fn: p = 0.0005; Student's t-test) (Fig 4B). HUVECs seeded on “High Fn” dishes showed a stronger calcium signaling in response to mechanical stimulation, followed by the cells on “Low Fn” dishes. Cells on “No Fn” dishes showed the weakest response. By calculating the relative difference for all mechanically stimulated cells under these three different conditions, there are statistically significant differences among all of them (High Fn and Low Fn: p = 0.04, High Fn and No Fn: p = 4x10^-7^, Low Fn and No Fn: p = 0.003; Student's t-test) (Fig 4C).

### TRPM7 siRNA increases adhesion of HUVEC but inhibits response to stimulation

**Fig 5:**
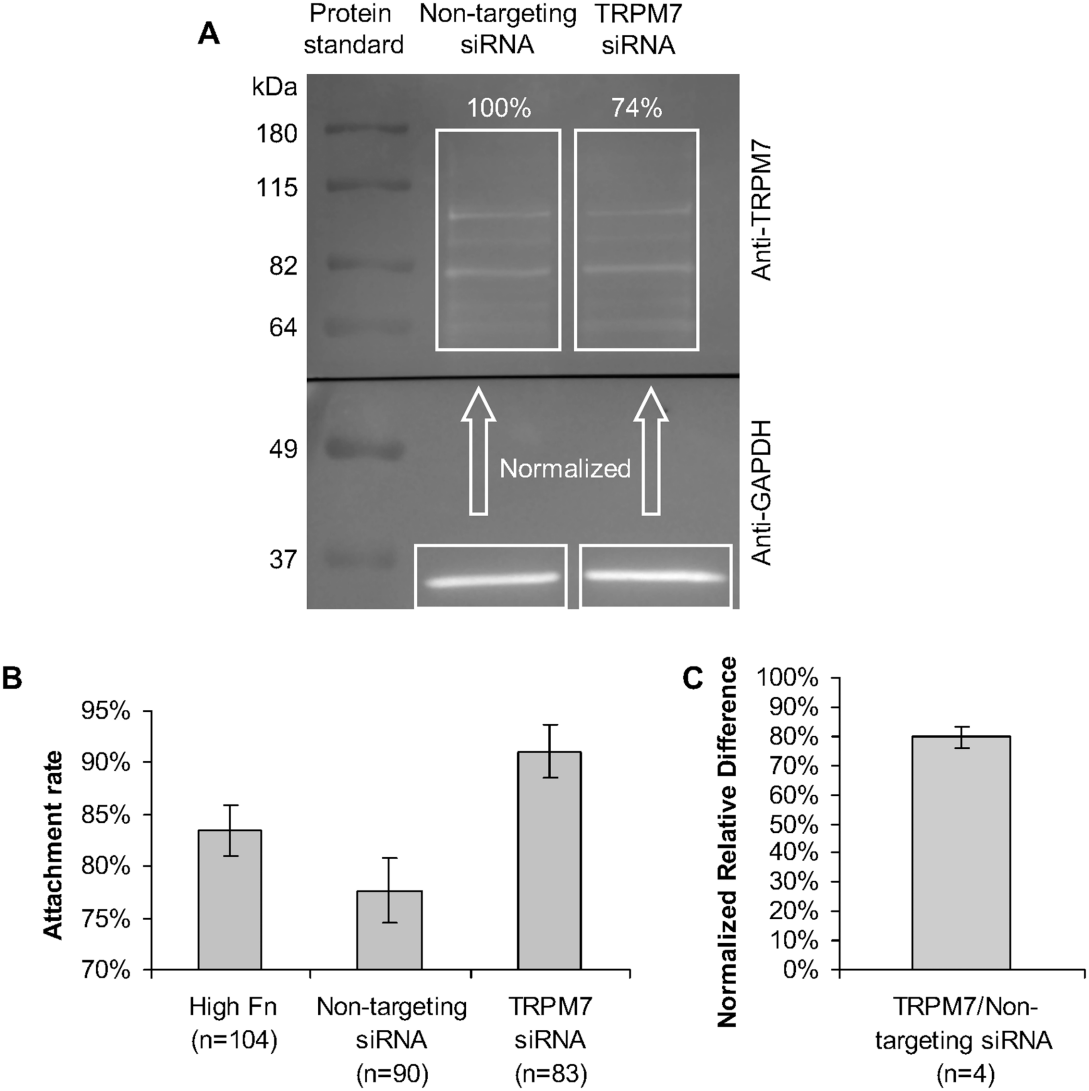
TRPM7 siRNA increases adhesion of HUVEC but inhibits response to stimulation. HUVEC electroporated with non-targeting siRNA and TRPM7 siRNA had their adhesion and calcium response to mechanical stimulation compared. (A) Attachment ratios of HUVECs electroporated with non-targeting and TRPM7 siRNAs. These results are also compared to a control, HUVECs on "High Fn" dishes. Control and non-targeting siRNA are the only ones not statistically different (control and non-targeting siRNA: p = 0.2, control and TRPM7 siRNA: p = 0.03, non-targeting and TRPM7 siRNAs: p = 0.001; Student's t-test) (B) Relative difference of the biosensor fluorescence ratio of mechanically stimulated HUVECs non-targeting and TRPM7 siRNAs. They are statistically different (non-targeting and TRPM7 siRNAs: p = 0.01; Student's t-test). The error bar on the bar graphs are the respective standard error of the samples.

The TRPM7 siRNA inhibited the TRPM7 expression to 74% according to one Western blot assay (Fig 5A). Considering some protein fragmentation and the fact that the anti-TRPM7 was polyclonal, the TRPM7 expression for each condition was the sum of all the bands between 60 and 180 kDa. TRPM7 inhibition by siRNA was verified with one sample of immunobloting. Non-targeting siRNA did not have detectable effects, in contrast to TRPM7 siRNA whose specificity has been demonstrated previously [32]. As TRPM7 presence interferes with adhesion for different cell types [20,22], an adhesion test was performed to verify the effect of TRPM7 siRNA. Although non-targeting siRNA did not cause significant adhesion changes comparing to control cells (p = 0.2; Student's t-test), TRPM7 siRNA significantly enhanced adhesion (control and TRPM7 siRNA: p = 0.03, non-targeting and TRPM7 siRNAs: p = 0.001; Student's t-test) (Fig 5B). However, TRPM7 siRNA led to a reduced response of calcium in HUVECs to mechanical stimulation. To deal with the noise engendered from the cell heterogeneity, a large number of cells were included. The average relative difference of calcium response for cells treated with TRPM7 siRNA (n = 42) was normalized by that from cells treated with non-targeting siRNA (n = 37) in each experiment day. This normalized response was calculated for each of the 4 experiment days, with around 10 cells tested in each condition. The results revealed that the average response of TRPM7 siRNA-treated HUVECs was 80±4% of that from cells treated by non-targeting siRNA. The p-value in this case is the probability of a normal distribution with the experimental average and standard deviation to have a normalized *RelDiff* higher than 100%. The data showed statistical significance as clearly indicate the involvement of TRPM7 in regulating cellular physiology (p = 3x10^-7^) (Fig 5C).

## Discussion

### Device is capable of delivering localized high temporal frequency mechanical stimulation

The mechanical stimulation delivered by the present setup allowed a deformation with temporal frequency high enough to potentially increase calcium response as previously reported [15]. Lower temporal frequencies of the same equipment in the order of 0.5~1 Hz did not cause significant calcium signaling (data not shown), highlighting the importance of the current equipment improvement in controlling stimulation frequency and delivering robust trigger. The frequency of vibration of the probe was only 6% below the estimated by the simplified model. As expected, this frequency was slightly below the estimated 220 Hz possibly due to the added mass (the probe tip) to the free end of the aluminum rod. The frequency of probe vibration did not seem to be related to the gel thickness in this particular case. Thus, it may suggest that the vertical displacement of the probe tip is not large enough to cause the probe interaction with the gel to be significantly different across the different gel thicknesses used. For this to occur, either the vertical displacement of the probe tip is small enough or the gel stiffness is low enough so that the effects of the elasticity and dampening of the gel substrate over the probe are the same for all thicknesses. The stimulation is also transient, as illustrated in Fig 2C and 2D. The vibration decays very quickly relative to the interval between the vibration trigger and the [Ca^2+^]_i_ response. During the image acquisition of calcium signaling, a few seconds after the stimulus was delivered, no vibration is observed anymore. The strain on the cell substrate caused by this design was slightly higher than other works involving cell injury [6,15], similarly to the previous design [28]. Considering the usual size and position of the cell relative to the probe tip position, the strain was larger at regions closer to the probe, which showed the localized nature of the stimulation delivered and was consistent to the region of stronger [Ca^2+^]_i_ rise in the observed responses. Thus, the localized nature of the stimulation and its high temporal frequency makes the setup very useful for studying calcium signaling in mechanotransduction, the main object of study in this work.

### The adhesion assay developed showed increased sensitivity and simplicity

The adhesion assay developed, with cell counting only occurring at the periphery of the gel, showed to be simple but effective in assessing the adhesion. The main disadvantage was the time needed to count the cells. Other methods involve biochemical assays, such as the Cell Proliferation Reagent WST-1 (Roche), which could be used but may result in lower sensitivity. Thus, the main advantages of our cell adhesion assessment technique were increased sensitivity and simplicity, as the assay did not require more than an orbital shaker and a microscope.

### Cell adhesion relationship to cell response was established to allow conclusions about the TRPM7 role in mechanotransduction

Even though TRPM7 is a known ion channel, it also has a kinase domain and its inhibition or overexpression does affect adhesion for some cell types [20,22]. In other words, modulating TRPM7 does not simply modulate the mechanosensitive ion channels but also the cell adhesion, which is related to the cell response by improving the force transmission from the substrate to the cell. Thus, to understand the role of TRPM7 in mechanotransduction, it is necessary to know the relationship between cell response and adhesion in our particular case. HUVECs seemed to be very adhesive and sensitive to adhesion molecules, showing calcium response to mechanical stimulation even with relatively little fibronectin coating: although “Low Fn” dishes had only one tenth of the fibronectin on “High Fn” dishes, the magnitude of the calcium response reported by the biosensor was still about 75% of the average from “High Fn” dishes. Although polyacrylamide gels were supposed to limit non-specific cell adhesions, HUVECs managed to attach to the substrate when no sulfo-SANPAH nor fibronectin were present, showing about 20% of the response from control. HUVECs seem to be quite adhesive, even adhering to unprepared surfaces like glass (not shown). The magnitude of response correlated to the trend of attachment of cells after the adhesion test, with the highest attachment ratio (83%) for the HUVECs seeded on the “High Fn” polyacrylamide gel with full treatment (sulfo-SANPAH and fibronectin), lower attachment ratio (71%) for the “Low Fn” gel covered with only 5% of the normal fibronectin amount (and no sulfo-SANPAH treatment) and the lowest attachment ratio (49%) for the “No Fn” untreated gel. Considering the case of an alteration in the proteins recruited by integrins due to fibronectin mobility differences between “High Fn” and “Low Fn” gels [33], there would be more adhesion sites detached from stress fibers on “Low Fn”. Thus, the overall transmission of forces to mechanosensitive channels expected for a given amount of integrins would be decreased. In this case, the possible effect of fibronectin mobility differences (not in the scope of this work) would not change the conclusions anyway. These results show the effectiveness of the different gel treatments for reducing the adhesion and the correlation between adhesion and cell response.

### TRPM7 channel function plays a stronger role in [Ca^2+^]_i_ mechanotransduction than adhesion modulation

To confirm that a separate analysis of mechanotransduction and adhesion is possible, it was necessary to assess the change in adhesion after TRPM7 was inhibited by siRNA. The results pointed to an increased attachment ratio (91%) when TRPM7 is inhibited when compared to both control (83%) and non-targeting siRNA-treated cells (78%). TRPM7 is colocalized with m-Calpain, which has a [Ca^2+^]_i_ dependency to disassemble focal adhesions [22]. As the cell adhesion was improved with TRPM7 inhibition, one would expect a stronger [Ca^2+^]_i_ response to mechanical stimulation due to an improved force transmission to the cell. However, the actual weaker response obtained by HUVEC with TRPM7 inhibition suggests that the TRPM7 channel functionality is indeed essential and important for the calcium signaling response to mechanical stimulation.

TRPM7 inhibition in fibroblasts disrupts the organization of actin stress fibers, myosin IIA and vinculin, a focal adhesion marker, by an unknown mechanism [34]. TRPM7 has been considered a stretch-activated cation channel (SACC), responsible for triggering calcium flickers, which are fast (10 milliseconds to 4 seconds) and spatially localized [Ca^2+^]_i_ spikes. These calcium flickers, then, steer cell migration [35]. On the other hand, TRPM7 colocalizes with invadosomes, which would suggest a calcium dependent modulation, but the calcium flickers do not colocalize with them. The TRPM7 modulation of invadosomes was reported to be independent of calcium influx for mouse neuroblastoma cells [36]. Thus, an investigation involving mechanical stimulation and calcium flickers would be interesting for clarifying the relationship between TRPM7 and migration/adhesion. Particularly in the present work, no calcium flickers were noticed as the usual period for image acquisition (around 30 seconds) was a lot larger than the calcium flicker lifetime. Also, the minimum image acquisition period was limited by the time necessary for the filters to change and for the exposure of each image, usually around 5 or more seconds, which would decrease the chance of observing a calcium flicker. Examples of [Ca^2+^]_i_ responses for all conditions are shown in S4 Video through S8 Video. Thus, provided a faster image acquisition is available, the present work describes a collection of techniques that may be useful for studying the relationship between TRPM7 mechanotransduction role and cell migration/adhesion.

In conclusion, this work proposes a combination of simple methods to study mechanotransduction with a localized high temporal frequency mechanical stimulation and a more sensitive adhesion assay. This combination was able to separate the effects of TRPM7 adhesion modulation from the overall cell response, showing the stronger role of TRPM7 in mechanotransduction in this case and this setup potential for other similar studies.

## Supporting Information

S1 Video Polyacrylamide substrate deformation during vibration stimulation. Video taken with a high-speed camera, at 1,000 frames per second. The polyacrylamide gel under deformation has 1 μm beads embedded close to the surface. The black circle represents the typical position of a cell (~125 μm of diameter) during stimulation.
(AVI)

S2 Video Polyacrylamide substrate deformation plotted as a colormap/vectormap. The deformation observed with the high-speed camera was utilized to calculate the spatial colormap of the displacement with vectors at different locations indicating the direction and magnitude at the local positions. The black circle represents the typical position of a cell (~125 μm of diameter) during stimulation. The bar represents 25 μm.
(AVI)

S3 Video Polyacrylamide substrate strain plotted as a colormap. The strain, calculated from the deformation observed with the high-speed camera, was plotted as a spatial colormap, with displacement displayed as vectors. The black circle represents the typical position of a cell (~125 μm of diameter) during stimulation. The bar represents 25 μm.
(AVI)

S4 Video Example of calcium response of cells in “High Fn” dishes upon mechanical stimulation. Video of the colormap in a cell representing [Ca^2+^]_i_ measured by the fluorescence emission ratio of YPet/ECFP from the calcium biosensor before and after the mechanical stimulation. The scale bar represents 25 μm.
(AVI)

S5 Video Example of calcium response of cells in “Low Fn” dishes upon mechanical stimulation. Video of the colormap in a cell representing [Ca^2+^]_i_ measured by the fluorescence emission ratio of YPet/ECFP from the calcium biosensor before and after the mechanical stimulation. The scale bar represents 25 μm.
(AVI)

S6 Video Example of calcium response of cells in “No Fn” dishes upon mechanical stimulation. Video of the colormap in a cell representing [Ca^2+^]_i_ measured by the fluorescence emission ratio of YPet/ECFP from the calcium biosensor before and after the mechanical stimulation. The scale bar represents 25 μm.
(AVI)

S7 Video Example of calcium response of cells treated with non-targeting siRNA upon mechanical stimulation. Video of the colormap in a cell representing [Ca^2+^]_i_ measured by the fluorescence emission ratio of YPet/ECFP from the calcium biosensor before and after the mechanical stimulation. The scale bar represents 25 μm.
(AVI)

S8 Video Example of calcium response of cells treated with TRPM7 siRNA upon mechanical stimulation. Video of the colormap in a cell representing [Ca^2+^]_i_ measured by the fluorescence emission ratio of YPet/ECFP from the calcium biosensor before and after the mechanical stimulation. The scale bar represents 25 μm.
(AVI)
