# Correction: Rapid and Localized Mechanical Stimulation and Adhesion Assay: TRPM7 Involvement in Calcium Signaling and Cell Adhesion

**DOI:** 10.1371/journal.pone.0138281

**Published:** 2015-09-14

**Authors:** Wagner Shin Nishitani, Adriano Mesquita Alencar, Yingxiao Wang

In the Data Availability section, there is an error in the DOI for the data uploaded to the Harvard Dataverse Network. The correct DOI is 10.7910/DVN/O239GQ. The complete, correct Data Availability section should read as follows:

All files are available from the Harvard Dataverse Network (http://thedata.harvard.edu/dvn/, doi: 10.7910/DVN/O239GQ).
